# Productive Infection of Bovine Papillomavirus Type 2 in the Urothelial Cells of Naturally Occurring Urinary Bladder Tumors in Cattle and Water Buffaloes

**DOI:** 10.1371/journal.pone.0062227

**Published:** 2013-05-07

**Authors:** Sante Roperto, Valeria Russo, Ayhan Ozkul, Annunziata Corteggio, Aylin Sepici-Dincel, Cornel Catoi, Iolanda Esposito, Marita G. Riccardi, Chiara Urraro, Roberta Lucà, Dora M. Ceccarelli, Michele Longo, Franco Roperto

**Affiliations:** 1 Dipartimento di Medicina Veterinaria e Produzioni Animali, Sezione Malattie Infettive, Università di Napoli Federico II, Naples, Italy; 2 Dipartimento di Medicina Veterinaria e Produzioni Animali, Sezione Patologia Generale, Università di Napoli Federico II, Naples, Italy; 3 Department of Pathology, Faculty of Veterinary Medicine, Ankara University, Ankara, Turkey; 4 Health Research and Practice Center, Faculty of Medicine, Gazi University, Ankara, Turkey; 5 Department of Pathology, Faculty of Veterinary Medicine, University of Agricultural Sciences and Veterinary Medicine, Cluj-Napoca, Romania; 6 Istituto di Endocrinologia e Oncologia Sperimentale “Gaetano Salvatore”, Consiglio Nazionale delle Ricerche, Naples, Italy; 7 Dipartimento di Biologia, Università di Napoli Federico II, Naples, Italy; Federal University of Pelotas, Brazil

## Abstract

**Background:**

Papillomaviruses (PVs) are highly epitheliotropic as they usually establish productive infections within squamous epithelia of the skin, the anogenital tract and the oral cavity. In this study, early (E) and late (L) protein expression of bovine papillomavirus type 2 (BPV-2) in the urothelium of the urinary bladder is described in cows and water buffaloes suffering from naturally occurring papillomavirus-associated urothelial bladder tumors.

**Methods and Findings:**

E5 protein, the major oncoprotein of the BPV-2, was detected in all tumors. L1 DNA was amplified by PCR, cloned and sequenced and confirmed to be L1 DNA. The major capsid protein, L1, believed to be only expressed in productive papillomavirus infection was detected by Western blot analysis. Immunohistochemical investigations confirmed the presence of L1 protein both in the cytoplasm and nuclei of cells of the neoplastic urothelium. Finally, the early protein E2, required for viral DNA replication and known to be a pivotal factor for both productive and persistent infection, was detected by Western blot and immunohistochemically. Electron microscopic investigations detected electron dense particles, the shape and size of which are consistent with submicroscopic features of viral particles, in nuclei of neoplastic urothelium.

**Conclusion:**

This study shows that both active and productive infections by BPV-2 in the urothelium of the bovine and bubaline urinary bladder can occur *in vivo*.

## Introduction

Papillomaviruses (PVs) are small oncogenic highly epitheliotropic DNA viruses showing a marked tropism for squamous epithelium [1]. Papillomaviruses are known to establish productive infections only within stratified epithelia of the skin, the anogenital tract and the oral cavity [2].

Among the thirteen bovine papillomavirus (BPV) types, (BPV-1 to -13), that have been completely described [3], [4], BPV-1/-2 are closely related types [5] and assigned to the genus Deltapapillomavirus, species 4, the biological properties of which include lesions in ungulates, fibropapillomas in the respective host and interspecies-transmission [2], [6]. BPV-1/-2 are the only papillomaviruses known to infect both epithelial and mesenchymal cells. Similar to other PVs, BPV-1/-2 replication and virion production appear to be confined to the epithelial region of the lesions [5]. The viral life cycle is tightly linked to the differentiation of the infected epithelial cells. It has been suggested that production of viral capsid proteins takes place in productive infections only [2], assembly of the virion particles takes place in the granular and cornified layers and virus particles are released as the dead squamous cells disintegrate [2], [7]. However, it has been recently shown that other cells such as peripheral blood mononuclear cells (PBMCs) and placental trophoblastic epithelium are additional sites of *in vivo* productive infection of BPV-2 [8], [9].

The E5 protein, the major oncoprotein of BPV-2, is responsible for cell transformation through different molecular pathways. It has been shown that in spontaneous bladder carcinogenesis of cattle E5 oncoprotein binds to the activated form of the platelet derived growth factor β receptor (PDGFβ-R) [10]. More recently, a new molecular mechanism via Calpain 3 activation has been studied in bovine urothelial tumors [11].

The papillomavirus E2 early protein is a pivotal factor of both productive and persistent infection as it is the main regulator of viral DNA replication and viral gene transcription [2], [12]. It has been suggested that high levels of E2 during the viral life cycle regulate the expression of the late papillomavirus genes L1 and L2 thus facilitating the new virus progeny [2], [13]. Novel biological activities of E2 have been recently proposed. It has been suggested that E2 plays an important role in intracellular trafficking as it interacts with a functional family of proteins involved in vesicle-mediated transport mostly between Golgi apparatus and endosomes as well as endosomes and lysosomes [12].

It is well-known that BPV-1/-2 play a role in bladder carcinogenesis of large ruminants as they can infect the urothelium of the urinary bladder in which they usually establish latent and/or abortive infections. BPV-2 has appeared to be responsible for bladder tumors of cattle for several years already [14], [15], [16]. Although it has been suggested that papillomavirus disease is still little-known in buffalo and urinary bladder pathology is a neglected area of investigations in this species [17], [18], however the BPV-2 infection of the urothelial cells of the urinary bladder resulting to urothelial tumors has very recently been shown to occur also in buffalo [19]. High-risk human papillomavirus (HPV) infection has been also proposed as responsible for some urothelial tumors in man [20].

The present report documents the expression of BPV-2 L1 protein and virion assembly in the urothelial cells of naturally occurring urinary bladder tumors. This study is the first to reveal that the neoplastic urothelium of the bovine and bubaline urinary bladder is an additional site of productive BPV-2 infection.

## Materials and Methods

### Ethics Statement

In this study we did not perform any animal experiments. We collected the samples directly from public slaughterhouses; the animals were slaughtered following owners' decisions and after a mandatory clinical *ante-mortem* examination, as required by the European Union legislation.

### Tissue samples

Neoplastic bladder samples were collected from twenty cows and twenty-one water buffaloes both at public and private Italian and Turkish slaughterhouses, respectively. As far as bovine samples are concerned they were collected, after the permission of the medical authorities, in the slaughterhouses named ‘Macello Comunale’ of Muro Lucano (PZ), ‘Fratelli Peta’ of Marcellinara (CZ) and ‘Barbara Rocco sas’ of Simbario (VV). As far as buffalo samples are concerned they were collected, after the permission of the medical authorities, in the slaughterhouses named ‘Bafra Belediye Mezbahas’ in Bafra/Samsun and ‘Coskun Et ve Mamülleri Sanayi ve Ticaret Anonim irketi et Fabrikas Kesimhanesi, Kagithane’ at Istanbul. To prevent possible cross-contaminations, each sample was immediately divided into several parts that were frozen in liquid nitrogen for subsequent molecular biological analysis, or fixed in 10% neutral buffered formalin and in 4% glutaraldehyde in 0.1 M phosphate buffer for microscopical and electron microscopical investigations.

### Histopathology

Tissues fixed in 10% neutral buffered formalin were routinely processed for paraffin embedding. Histologic diagnosis of bladder tumors was assessed on 5-µm-thick hematoxylin-eosin (HE)-stained sections.

### BPV-2 E5 Immunoprecipitation

Tissue samples from urinary bladders of cows and water buffaloes were lysed in ice-cold buffer containing 50 mM Tris-HCl (pH 7.5), 1% (v/v) Triton X-100, 150 mM NaCl, 2 mM PMSF, 1.7 mg/ml Aprotinin, 50 mM NaF, and 1 mM sodium orthovanadate. The protein concentration was measured using the Bradford assay (Bio-Rad Laboratories, Milan, Italy). Proteins derived from bladders (1000 µg) were immunoprecipitated by using 2 µg of a polyclonal sheep anti-E5 antibody (a kind gift by Dr. M.S. Campo) and 30 µl of Protein A/G-Plus Agarose (Santa Cruz Biotechnology, CA, USA). Immunoprecipitates were washed five times in complete lysis buffer (above), finally heated in 1X Laemmli sample buffer at 100°C for 10 min. Immunoprecipitates were subjected to sodium dodecyl sulfate–polyacrylamide gel electrophoresis (SDS–PAGE) (15% polyacrylamide) under reducing conditions. After electrophoresis, proteins were transferred on nitrocellulose filter membrane (GE Healthcare Life Sciences, Chalfont St Giles, UK) for 1 h at 300 mA in 192 mM glycine/25 mM Tris-HCl (pH 7.5)/10% methanol. Membranes were blocked for 1 h at room temperature in 5% non-fat dry milk, incubated with anti-E5 antibody overnight at 4°C. After three washes in Tris-buffered saline, membranes were incubated with rabbit anti-sheep IgG-horseradish peroxidase (HRP) (Santa Cruz Biotechnology, CA, USA) for 60 min at room temperature. Proteins were visualized by enhanced chemiluminescence system (Western Blotting Luminol Reagent, Santa Cruz Biotechnology, CA, USA).

### BPV-2 L1 DNA detection, cloning and sequencing

DNA was extracted from urinary bladder samples from 20 cows and 21water buffaloes with urothelial bladder tumors and from five healthy cows and five water buffaloes using the DNeasy Tissue Kit (Qiagen) according to the manufacturer's protocol. All the samples were lysed using proteinase K. Lysates were loaded onto DNeasy spin columns. After two washings pure DNA was eluted in low salt buffer. PCR analysis was performed using specific primers for BPV-2, L1 region: nt 1318±1341, 5′-GTTATACCACCCAAAGAAGACCCT-3, and nt 1490±1466, 5′-CTGGTTGCAACAGCTCTCTTTCTC-3′ [21]. To evaluate the adequacy of the DNA samples, a control PCR for bovine β-actin sequence was performed using a set of primers designed by Primer BLAST software (forward, 5′-GAGCGTGGCTACAGCTTCAC-3′; reverse, 5′-CATTGCCGATGGTGATGA-3′). Aliquots 50–100 ng of purified DNA were amplified in 25 µl of reaction mixture containing 1.5 mM MgCl_2_ for β-actin primers and 2 mM for BPV-2 L1 primers, 200 mM each dNTP, 480 nM of each primer and 2.5 U of AmpliTaq Gold DNA Polymerase (Applied Biosystems, Monza, Italy). The reaction was carried out in a thermocycler (Veriti, Applied Biosystems) with an initial denaturation step of 3 min. Then, 35 cycles of amplification were carried out with a denaturation step at 94°C for 40 sec, an annealing step at 60°C, 30 sec, for β-actin or at 55°C,40 sec, for BPV-2 L1, and an extension step at 72°C for 1 min. A final extension step at 72°C for 7 min was performed in each PCR assay. Detection of the amplified products was carried out by electrophoresis on ethidium bromide-stained agarose gel. In each experiment, a blank sample consisting of reaction mixture without DNA and a positive sample consisting of cloned BPV-2 (a kind gift by Dr. A. Venuti) were included. The quality of DNA was tested with primers for bovine β-actin gene. To confirm the PCR data, the amplified products were excised from the gel and purified through silicagel membranes by using the QIAquick PCR quantification kit according to the manufacturer's instructions (Qiagen, Milan, Italy). Then, the amplified DNA was cloned into pGEM-T vector using the pGEM®-T Easy Vector Systems (Promega, Milan, Italy), and sequenced in an automated apparatus (ABI PRISM 3100 Genetic Analyzer, Applied Biosystems, Monza, Italy).

### Western blot analysis for L1 and E2

Samples were solubilized at 4°C in lysis buffer containing 50 mM Tris-HCl pH 7.5, 150 mM NaCl, 1% Triton X-100. Immediately prior to use, the following reagents were added: 1 mM DTT, 2 mM PMSF, 1.7 mg/ml Aprotinin, 25 mM NaF, 1 mM Na3VO4 (Sigma-Aldrich, Milan, Italy).

Lysates were clarified at 21,500×*g* for 30 min. The protein concentration was measured using the Bradford assay (Bio-Rad Laboratories, Milan, Italy). For Western blotting, 50 µg of lysate proteins were heated at 100°C in 4X premixed Laemmli sample buffer. Proteins were separated on polyacrylamide gel and transferred to nitrocellulose filter membrane (GE Healthcare Life Sciences, Chalfont St Giles, UK) for 1 h at 350 mA in 192 mM glycine/25 mM Tris-HCl (pH 7.5)/10% methanol. The membranes were blocked with 5% non-fat dry milk in Tris-buffered saline (TBS, pH 7.5) for 1 h at room temperature, washed with TBS-0.1% Tween. Then, the membranes were probed with a monoclonal mouse anti-HPV-16 L1 (Chemicon International, CA, USA) and a polyclonal rabbit anti-E2 (a kind gift by Dr. E. Androphy) antibodies for an overnight incubation at 4°C. After three washes in Tris-buffered saline, membranes were incubated with horseradish peroxidase-conjugated anti-rabbit IgG (Bio-Rad Laboratories, Milan, Italy) for 1 h at room temperature. After three washing steps, bound antibody was visualized by an enhanced chemiluminescence system (Western Blotting Luminol Reagent, Santa Cruz Biotechnology, CA, USA).

### Immunohistochemistry

Neoplastic and normal bladder sections were processed with the same procedures. Briefly, the sections were deparaffinized and then endogenous peroxidase activity was blocked by incubation in 0.3% H_2_O_2_ in methanol for 20 min. Antigen retrieval was performed by pretreating with microwave heating (twice for 5 min each at 750 W) in citrate buffer pH 6.0. The slides were washed three times with phosphate buffered saline (PBS), pH 7.4, 0.01 M, then incubated for 1 h at room temperature with rabbit serum (Sigma-Aldrich, Milan, Italy) diluted at 1 in 10 in PBS. The excess serum was drained off and a polyclonal sheep anti-BPV-2 E5 primary antibody (a kind gift by Dr. M.S. Campo) diluted at 1 in 40,000 in PBS, was applied for 1 h at room temperature in a humid chamber. Following incubation, the sections were rinsed three times for 5 min with PBS before application of the rabbit anti-sheep biotinylated secondary antibody (Santa Cruz Biotechnology, Inc., CA, USA), diluted at 1 in 100 in PBS for 45 min at room temperature. For E2 and L1 detection the slides were washed three times with PBS, pH 7.4, 0.01 M, then incubated for 1 h at room temperature with protein block serum-free (DakoCytomation, Denmark). The anti-BPV-2 E2 primary antibody diluted at 1 in 50/100 in PBS and the anti-HPV-16 L1 diluted at 1 in 200 in PBS were applied overnight at 4°C in a humid chamber. The sections were rinsed three times for 5 min with PBS, incubated for 40 min at room temperature with appropriate biotinylated secondary antibody (labelled streptavidin-biotin (LSAB) Kit; DakoCytomation, Denmark). Finally, all the sections were washed three times with PBS and then incubated with streptavidin-conjugated to horseradish peroxidase (LSAB Kit; DakoCytomation, Denmark). Color development was obtained by treatment with diaminobenzidine (DakoCytomation, Denmark) for 5–20 min. Sections were counter stained with Mayer's hematoxylin.

### Transmission Electron Microscopy

Ten bladder samples from cattle and twelve from water buffaloes, were immediately fixed in 4% glutaraldehyde in 0.1 M phosphate buffer (pH 7.4) for 2–3 h. They were washed (20 min 5 times) and post fixed in 1% OsO4 in the same buffer for 1 h. They were washed again in 0.1 M phosphate buffer (pH 7.4) and then dehydrated in graded alcohol, and embedded in Agar Low Viscosity Resin (Agar Scientific Limited, Essex, England). Semi-thin section (400 nm) were cut on an EM UC6 ultramicrotome (Leica Microsystems) and were stained with 1% toluidine blue in water solution and examined by light microscopy. Ultra thin sections (60–70 nm), obtained from chosen areas, were collected onto 300-mesh grids coated with formvar and counterstained with lead citrate and uranyl acetate. The sections were observed with a JEOL JEM-1011 transmission electron microscope (JEOL, Tokyo, Japan) equipped with a thermionic tungsten filament and operated at an acceleration voltage of 100 kV. Images were taken using a Morada cooled slow-scan CCD camera (3783×2672 pixels) and micrographs were taken with iTEM software (Olympus Soft Imaging System GmbH, Munster, Germany). The same procedure was used to obtain ultra thin sections from normal bladder of healthy cows and water buffaloes.

## Results

**Figure 1:**
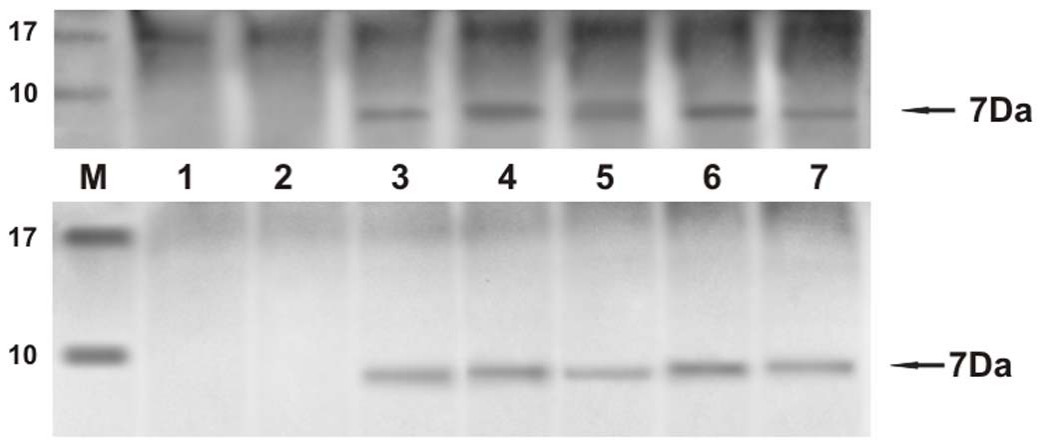
E5 immunoprecipitation. The presence of E5 protein detected by immunoprecipitation. a) Lanes 1–2: urinary bladders from healthy cows. Lanes 3–7: five tumors of the urinary bladders in cows. b) Lanes 1–2: urinary bladders from healthy buffaloes. Lanes 3–7: five tumors of the urinary bladders in buffaloes.

**Figure 2:**
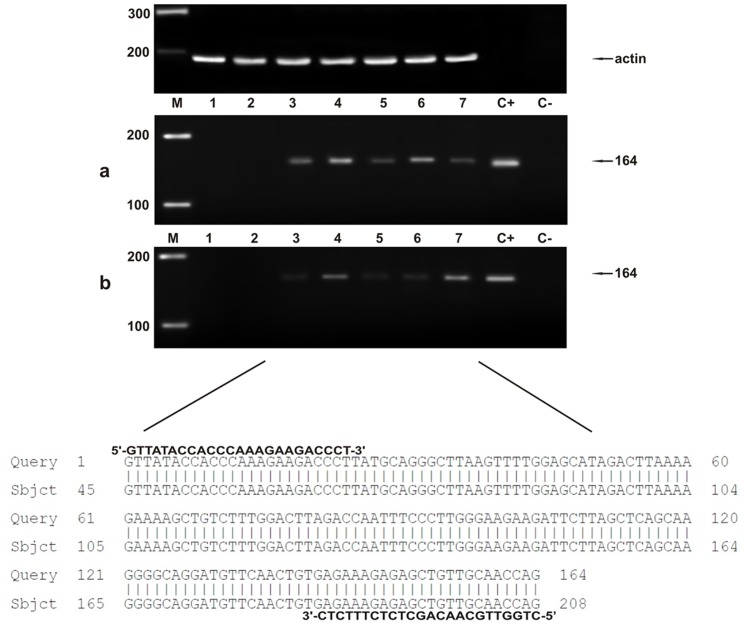
PCR amplification of DNA samples. a) Lane M, molecular mass marker (HyperLadder II Bioline); lanes 1–2 urinary bladder samples from healthy cows without BPV-2 L1 DNA; lanes 3–7 bladder tumors samples from five of twenty cows showing BPV-2 L1 DNA; lane C+, positive control (cloned BPV-2 DNA); lane C-, negative control (no DNA added). The arrow indicates the position of the 164 bp BPV-2 L1 PCR product. b) Lane M, molecular mass marker (HyperLadder II Bioline); lanes 1–2 urinary bladder samples from healthy buffaloes without BPV-2 L1 DNA; lanes 3–7 bladder tumors samples from five of twenty-one buffaloes showing BPV-2 L1 DNA; lane C+, positive control (cloned BPV-2 DNA); lane C-, negative control (no DNA added). The arrow indicates the position of the 164 bp BPV-2 L1 PCR product. The lower part of the figure shows 100% homology between the sequence of the amplicons in lanes 3–7 and the sequence of BPV-2 L1 found in Italy (GenBank M20219).

Table 1 and Table 2 report histological diagnosis of urothelial tumors of the urinary bladder of cattle and buffaloes, respectively, performed according to recent morphological criteria [22]. In all tumor cases, the BPV-2 E5 oncoprotein was detected by immunoprecipitation (Figure 1). As it has been shown that BPV-2 plays a crucial role in bladder carcinogenesis of large ruminants [14], [19] and since the cattle and buffaloes affected with urothelial tumors, here reported, were from Italy and Turkey respectively, we analyzed the sequence of the L1 gene, the most conserved PV gene, to assess any variation in BPV-2 between the two countries. PCR analysis was performed in all bovine and bubaline samples. L1 DNA was amplified and a band of ∼55 kD was shown both in benign and malignant tumors (Figure 2). Amplicon sequencing detected a DNA fragment composed of 164 bp (Figure 2) showing an absolute homology (100%) with the known sequences of BPV-2 L1 DNA. There were no differences in L1 DNA sequence between cattle and buffaloes. GenBank accession number of our L1 sequence is M20219.

**Table 1:** Tumor histotypes and expression of viral proteins in urinary bladder neoplasias of cattle.

Samples	Microscopic patterns of tumors	BPV-2 E5	BPV-2 L1
T1	Papilloma	IP	WB
T2	Papilloma	IP	WB
T3	Papillary Urothelial Neoplasm of Low Malignant Potential	IP	WB
T4	Papillary Urothelial Neoplasm of Low Malignant Potential	IP	WB
T5	Low-Grade Papillary Urothelial Carcinoma	IP	WB
T6	Low-Grade Papillary Urothelial Carcinoma	IP	WB
T7	Low-Grade Papillary Urothelial Carcinoma	IP	WB
T8	Low-Grade Papillary Urothelial Carcinoma	IP	WB
T9	Low-Grade Papillary Urothelial Carcinoma	IP	WB
T10	Low-Grade Papillary Urothelial Carcinoma	IP	WB
T11	High-Grade Papillary Urothelial Carcinoma	IP	WB
T12	High-Grade Papillary Urothelial Carcinoma	IP	WB
T13	High-Grade Papillary Urothelial Carcinoma	IP	WB
T14	High-Grade Papillary Urothelial Carcinoma	IP	WB
T15	Low Grade Invasive Urothelial Carcinoma	IP	WB
T16	Low Grade Invasive Urothelial Carcinoma	IP	WB
T17	Low Grade Invasive Urothelial Carcinoma	IP	WB
T18	High-Grade Invasive Urothelial Carcinoma	IP	WB
T19	High-Grade Invasive Urothelial Carcinoma	IP	WB
T20	High-Grade Invasive Urothelial Carcinoma	IP	WB

**IP:** protein detection by immunoprecipitation; **WB:** protein expression shown by Western Blot.

**Table 2:** Tumor histotypes and expression of viral proteins in urinary bladder neoplasias of buffalo.

Samples	Microscopic patterns of tumors	BPV-2 E5	BPV-2 L1
T1	Papilloma	IP	WB
T2	Papilloma	IP	WB
T3	Papillary Urothelial Neoplasm of Low Malignant Potential	IP	WB
T4	Papillary Urothelial Neoplasm of Low Malignant Potential	IP	WB
T5	Low-Grade Papillary Urothelial Carcinoma	IP	WB
T6	Low-Grade Papillary Urothelial Carcinoma	IP	WB
T7	Low-Grade Papillary Urothelial Carcinoma	IP	WB
T8	Low-Grade Papillary Urothelial Carcinoma	IP	WB
T9	Low-Grade Papillary Urothelial Carcinoma	IP	WB
T10	Low-Grade Papillary Urothelial Carcinoma	IP	WB
T11	High-Grade Papillary Urothelial Carcinoma	IP	WB
T12	High-Grade Papillary Urothelial Carcinoma	IP	WB
T13	High-Grade Papillary Urothelial Carcinoma	IP	WB
T14	High-Grade Papillary Urothelial Carcinoma	IP	WB
T15	Low Grade Invasive Urothelial Carcinoma	IP	WB
T16	Low Grade Invasive Urothelial Carcinoma	IP	WB
T17	Low Grade Invasive Urothelial Carcinoma	IP	WB
T18	High-Grade Invasive Urothelial Carcinoma	IP	WB
T19	High-Grade Invasive Urothelial Carcinoma	IP	WB
T20	High-Grade Invasive Urothelial Carcinoma	IP	WB

**IP:** protein detection by immunoprecipitation; **WB:** protein expression shown by Western Blot.

**Figure 3:**
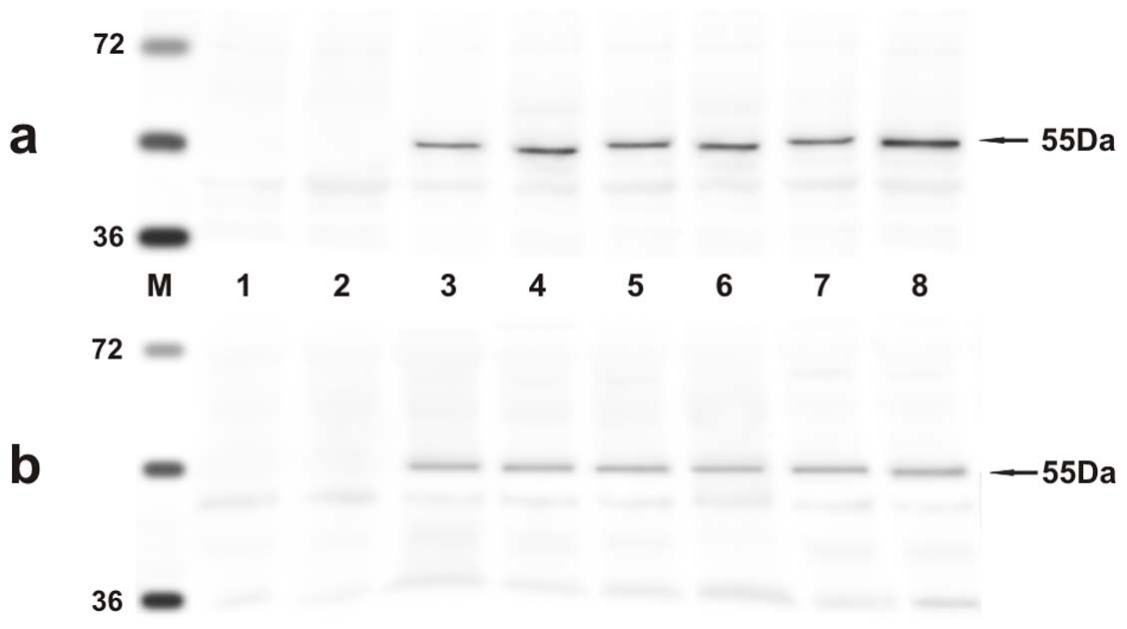
L1 expression. The presence of L1 protein detected by Western blot. a) Lanes 1–2: urinary bladders from healthy cows. Lanes 3–7: urinary bladder tumors from five of twenty cows. Lane 8: positive control, (bovine placenta infected with BPV-2). b) Lanes 1–2: urinary bladders from healthy buffaloes. Lanes 3–7: urinary bladder tumors from five of twenty-one buffaloes. Lane 8: positive control (bovine placenta infected with BPV-2).

**Figure 4:**
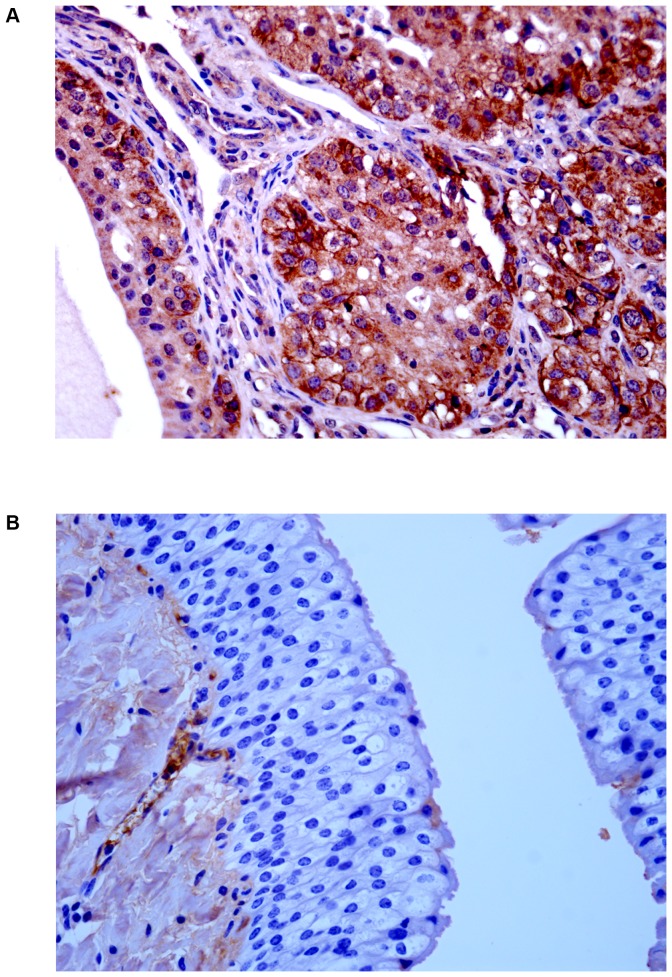
L1 immunohistochemistry. A) Immunohistochemical evidence of L1 protein in urothelial neoplastic cells. B) No L1 protein immunoreactivity was detected in normal urothelial cells.

Next, we assessed whether the L1 gene was expressed. We studied the expression of L1 across the spectrum of urothelial neoplastic lesions as it is well known that L1 expression reflects type and grade of tumors [23]. The presence of L1 protein in urothelial tumors of both cows and buffaloes was revealed by Western blot analysis (Figure 3). Its presence was confirmed morphologically. Immunohistochemically, a marked immunoreactivity was evident both in the cytoplasm and nuclei of the urothelial cells of neoplastic nests. Surprisingly, L1 protein expression was also evident in the basal neoplastic urothelial cells lining microcysts containing pale pink secretions (Figure 4A). No L1 protein expression was detected in normal urothelial cells (Figure 4B).

**Figure 5:**
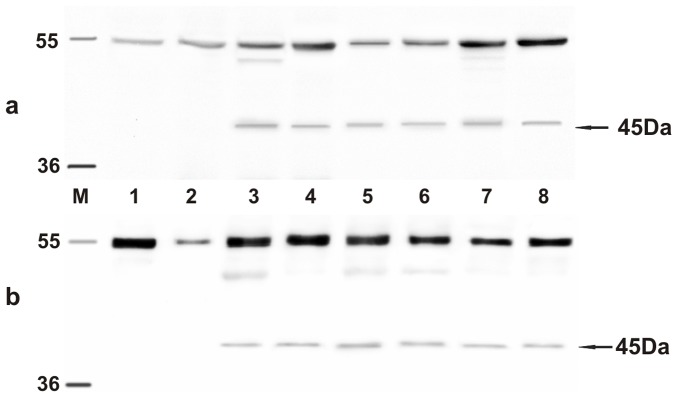
E2 expression. The presence of E2 protein detected by Western blot. a) Lanes 1–2: urinary bladders from healthy cows. Lanes 3–7: urinary bladder tumors from five of twenty cows. Lane 8: positive control, (bovine placenta infected with BPV-2). b) Lanes 1–2: urinary bladders from healthy buffaloes. Lanes 3–7: urinary bladder tumors from five of twenty-one buffaloes. Lane 8: positive control, (bovine placenta infected with BPV-2).

**Figure 6:**
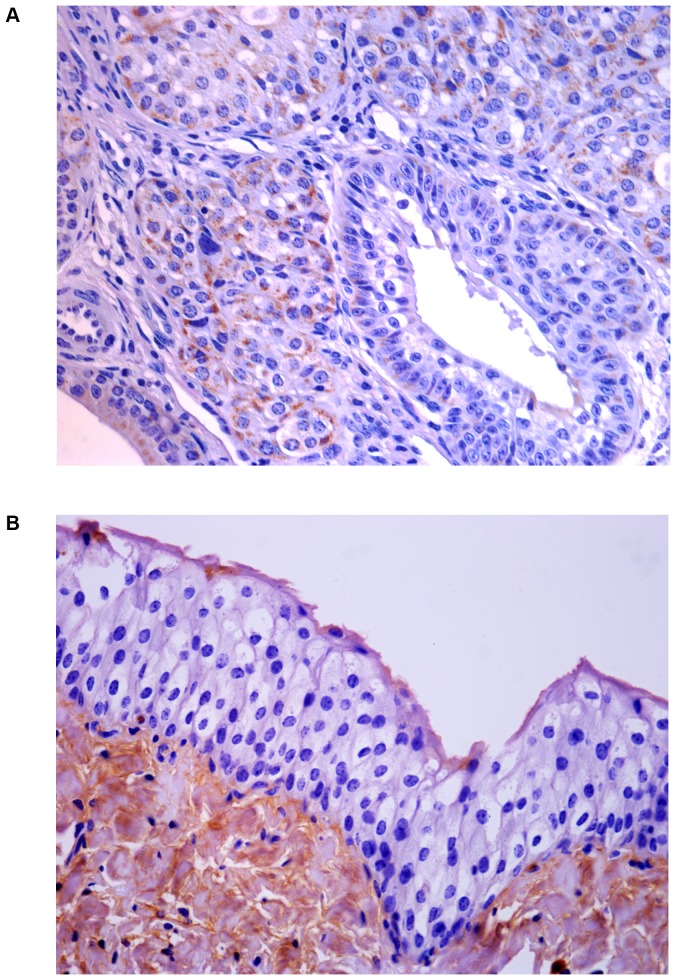
E2 immunohistochemistry. A) Immunohistochemical evidence of E2 protein in urothelial neoplastic cells. B) No E2 protein immunoreactivity was detected in normal urothelial cells.

Since it is known that E2 protein is essential for the viral life cycle and plays a part in productive infection, molecular and immunohistochemical studies on the expression of this protein were also carried out. E2 protein expression was detected by Western blot analysis in total protein extracts from both bovine and bubaline urothelial tumors (Figure 5). E2 protein expression was also shown with morphological procedures. Immunohistochemical studies confirmed the presence of E2 protein in the cytoplasm and nuclei of neoplastic urothelial cells (Figure 6A). E2 protein expression was not detected in normal urothelial cells (Figure 6B).

**Figure 7:**
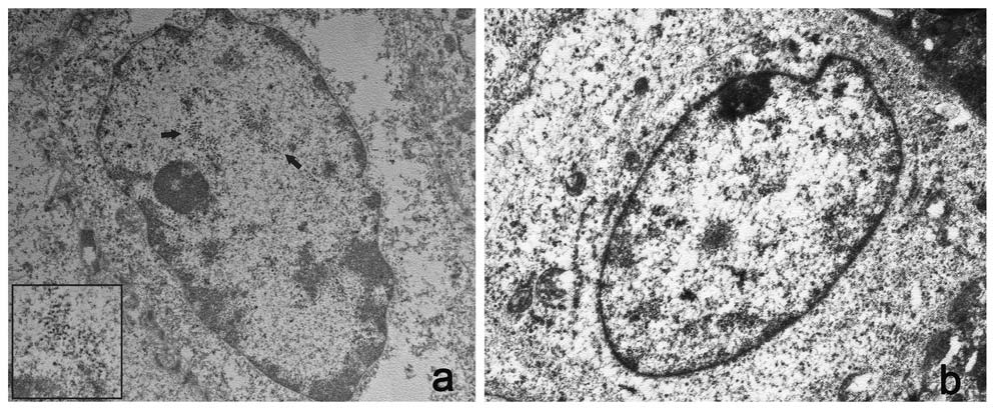
Presence of viral particles in bovine urothelial cells. A) Electron microscopical examination showed numerous intranuclear electron dense particles, 45–50 nm in diameter (black arrows). X 15,000. The size and shape of these particles are consistent with the submicroscopic features of viral particles (insert). X 50,000. B) **Normal urothelial cells.** No electron dense particles are detected in the nucleus of a normal urothelial cell. X 15,000.

**Figure 8:**
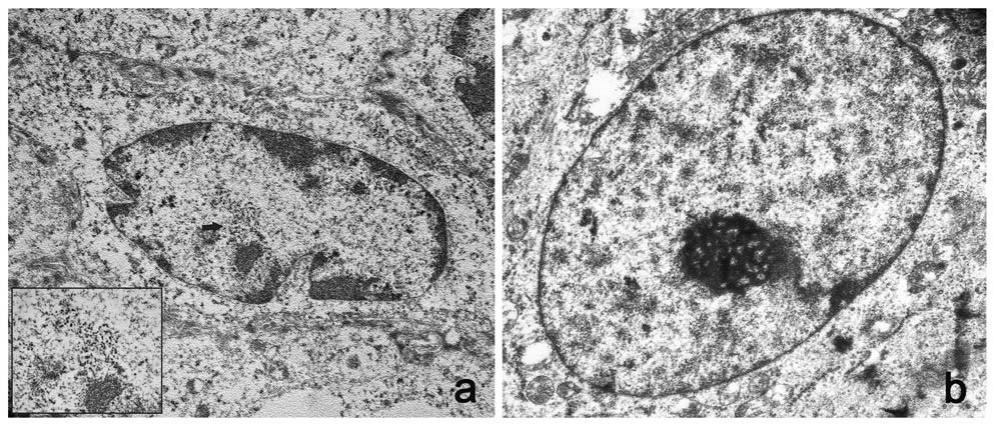
Presence of viral particles in bubaline urothelial cells. A) Numerous electron dense particles, 45–50 nm in diameter (black arrows) were seen in the nuclei. X 15,000. The size and shape of these particles are consistent with the submicroscopic features of viral particles (insert). X 50,000. B) **Normal urothelial cells.** No electron dense particles are seen in the nucleus of a normal urothelial cell. X 15,000.

Ultrastructural studies showed electron dense particles, about 50 nm in diameter, the size and shape of which were consistent with the submicroscopic features of viral particles both in the nuclei of bovine and bubaline urothelial cells (Figure 7A and 8A). Similar electron dense particles were not observed in the nuclei of urothelial cells from urinary bladders of healthy cows and buffaloes (Figure 7B and 8B).

## Discussion

It has been suggested that papillomavirus L1 protein, the major late capsid protein, is only expressed in productive infections and its presence is considered proof of a complete virus life cycle [2], [23]. Its expression depends on the episomal maintenance or the integration of viral genome into the host cell chromosome. In the case of human papillomavirus, and particularly HPV-16, the virus responsible for cervical cancer, the viral DNA needs to exist episomally in order to be productively replicated [24] and many cancer cells represent a “dead end” for the virus when viral DNA becomes integrated into the host cell chromosome, thus failing to produce infectious virions [25]. PV integration is considered to be a key event in the progression of persistent virus infection to invasive cancer [26], [27]. It is believed that viral integration could facilitate the accumulation of secondary genetic changes in host cell genome via oncoprotein expression. Genetic mutations and increased oncogene expression mediated by E2 are considered to be crucial events in the development of invasive cancer [24], [28]. However, it has recently been shown that integration of PV genome may cause no significant increase in oncoprotein expression following E2-disruption; furthermore it is still a matter of debate whether PV DNA integration leads to genomic instability or rather is a consequence thereof [29]. Very recently, it has been shown that over 60% of the cervical cancer cases harbor intact E2 despite the fact that PV E2 protein negatively regulates transcription of PV oncogenes [30].

L1 protein expression has been detected in high percentage in papillomavirus-associated pathological conditions such as inflammation and low grade lesions; it has been found to be very rarely expressed in high grade lesions and in cancers [26], [31], [32]. It has been suggested that L1 protein expression tends to be turned down with increasing severity of lesions as progression of low grade intraepithelial lesions to cancer appears to be associated with corresponding reduction of L1 protein expression [33], [34]. It is believed that the higher the malignant potential of tumor cells, the lower the expression of the L1 protein, until the viral genome becomes integrated and L1 is not detectable anymore [26], [35]. Therefore, it has been suggested that the detection of L1 protein could serve as possible prognostic marker useful in predicting the biological behavior of tumors [23], [33], [34].

Similar to the situation in cervical cancer, L1 expression appears to take place in typical papillomavirus-associated urothelial papilloma of the urinary bladder in man in which the state of the virus is known to be prevalently episomal [36] but it is not expressed in urothelial carcinoma, in whose cells the virus is known to occur integrated [20].

Tumors of the urinary bladder are common in cattle and buffaloes reared on pastures rich in bracken fern [19], [22], [37], [38]. Although an active papillomavirus infection is known to occur in the great majority of urothelial cancer cases in ruminants, neither structural viral antigens nor production of virus have ever been detected in bovine or bubaline bladder tumors [2], [19].

Our current molecular, microscopic and submicroscopic investigations provide for the first time evidence that a productive infection of BPV-2 occurs in the urothelial cells of cows and buffaloes suffering from naturally acquired BPV-2-induced bladder tumors.

We have detected the expression of BPV-2 E2 protein in urothelial cancer cells. It is known that E2 protein is necessary for continued maintenance of the viral episome and can enhance encapsidation of the viral genome into infectious particles thus playing a part in a productive infection [24], [39]. Furthermore, the expression of BPV-2 L1 protein strongly indicates that a complete life cycle of BPV-2 occurs also both in bovine and bubaline neoplastic urothelial cells. Up to now it has been believed that urothelial tumor cells were not permissive for productive infection by BPV-2. It has been suggested that the permanent episomal state of viral DNA could establish an abortive infection responsible for bladder carcinogenesis in cattle [37], [40]. However, we do not exclude that a greater quantity of E2 overexpression-mediated episomal BPV-2 may take place in urothelial cells which could result in increased expression of viral oncogenes thus exposing urothelial cells to a greater quantity of BPV-2 oncoproteins. Similar mechanisms have just been suggested for anal intraepithelial neoplasia (AIN) in human medicine [41].

Further studies are needed to know the infectivity of the viral particles and to better understand whether and/or how L1 protein, known to play a crucial role in infection and immunogenicity [42], stimulates the adaptive immune responses thus contributing to the chronic inflammation constantly seen in the stroma of bovine urothelial tumors [22], [43]. The interrelationship between tumor cells and their inflammatory microenvironments is a matter of debate [44]. Naturally occurring bladder carcinogenesis is quite common in cattle; therefore, we suggest this spontaneous tumor is an appropriate biological model for future research into papillomavirus-associated carcinogenesis.

## References

[1] Moody CA, Laimins LA (2010) Human papillomavirus oncoproteins: pathways to transformation. Nat Rev Cancer 10: 550–556. 20592731 10.1038/nrc2886

[2] IARC (2007) IARC Monographs on the Evaluation of Carcinogenic Risks to Humans. Vol 90, Human Papillomaviruses. Lyon, France: IARC Press. PMC4781057 18354839

[3] Zhu W, Dong J, Shimizu E, Hatama S, Kadota K, et al (2012) Characterization of novel bovine papillomavirus type 12 (BPV-12) causing epithelial papilloma. Arch Virol 157: 17–27. 10.1007/s00705-011-1140-7 22033594

[4] Lunardi M, Alfieri AA, Otonel RA, de Alcântara BK, Rodrigues WB, et al (2013) Genetic characterization of a novel bovine papillomavirus member of the Deltapapillomavirus genus. Vet Microbiol 162: 207–213. 22999523 10.1016/j.vetmic.2012.08.030

[5] Shafti-Keramat S, Schellenbacher C, Handisurya A, Christensen N, Reininger B, et al (2009) Bovine papillomavirus type 1 (BPV1) and BPV2 are closely related serotypes. Virology 393: 1–6. 19729180 10.1016/j.virol.2009.07.036 PMC3792341

[6] de Villiers ET, Fauquet C, Broker TR, Bernard HU, zur Hausen H (2004) Classification of papillomaviruses. Virology 324: 17–27. 15183049 10.1016/j.virol.2004.03.033

[7] Graham SV (2006) Late events in the life cycle of human papillomavirus. In: Campo MS, editor. Papillomavirus Research - from Natural History to Vaccines and Beyond, pp 193–211, Norfolk, UK: Caister Academic Press.

[8] Roperto S, Comazzi S, Ciusani E, Paolini F, Borzacchiello G, et al (2011) PBMCs are additional sites of productive infection of bovine papillomavirus type 2. J Gen Virol 92: 1787–1794. 21525209 10.1099/vir.0.031740-0

[9] Roperto S, Borzacchiello G, Esposito I, Riccardi M, Urraro C, et al (2012) Productive infection of bovine papillomavirus type 2 in the placenta of pregnant cows affected with urinary bladder tumors. PLoS One 7 (3) e33569 doi:10.1371/journal.pone.0033569. 22479413 10.1371/journal.pone.0033569 PMC3313941

[10] Borzacchiello G, Russo V, Gentile F, Roperto F, Venuti A, et al (2006) Bovine papillomavirus E5 oncoprotein binds to the activated form of the platelet-derived growth factor beta receptor in naturally occurring bovine urinary bladder tumours. Oncogene 25: 1251–1260. 16205631 10.1038/sj.onc.1209152

[11] Roperto S, De Tullio R, Raso C, Stifanese R, Russo V, et al (2010) Calpain3 is expressed in a proteolitically active form in papillomavirus-associated urothelial tumors of the urinary bladder in cattle. PLoS One 5 (4) e10299 doi: 10.1371/journal.pone.0010299. 20421977 10.1371/journal.pone.0010299 PMC2858658

[12] Muller M, Jacob Y, Jones L, Weiss A, Brino L, et al (2012) Large scale genotype comparison of human papillomavirus E2-host interaction networks provides new insights for E2 molecular functions. PLoS Pathog 8 (6) e1002761 doi:10.1371/journal.ppat.1002761. 22761572 10.1371/journal.ppat.1002761 PMC3386243

[13] Johansson C, Somberg M, Li X, Backström Winquist E, Fay J, et al (2012) HPV-16 E2 contributes to induction of HPV-16 late gene expression by inhibiting early polyadenylation. EMBO J 31: 3212–3227. 22617423 10.1038/emboj.2012.147 PMC3400011

[14] Roperto S, Brun R, Paolini F, Urraro C, Russo V, et al (2008) Detection of bovine papillomavirus type 2 in the peripheral blood of cattle with urinary bladder tumours: a possible biological role. J Gen Virol 89: 3027–3033. 19008389 10.1099/vir.0.2008/004457-0

[15] Campo MS (2006) Bovine papillomavirus: old system, new lessons? In: Campo MS, editor. Papillomavirus Research - from Natural History to Vaccines and Beyond, pp 373–387, Norfolk, UK:Caister Academic Press.

[16] Pathania S, Dhama K, Saikumar G, Shahi S, Somvanshi R (2012) Detection and quantification of bovine papilloma virus type 2 (BPV-2) by Real-PCR in urine and urinary bladder lesions in enzootic bovine haematuria (EBH)-affected cows. Transbound Emerg Dis 59: 79–84. 21797988 10.1111/j.1865-1682.2011.01248.x

[17] Somvanshi R (2011) Papillomatosis in buffaloes: a less-known disease. Transbound Emerg Dis 58: 327.332. 21435195 10.1111/j.1865-1682.2011.01211.x

[18] Somvanshi R, Pathania S, Nagarajan N, Pangty K, Kamar P (2012) Pathological study of non-neoplastic urinary bladder lesions in cattle and buffaloes: a preliminary report. Trop Anim Health Prod 44: 855–861. 21935661 10.1007/s11250-011-9978-y

[19] Roperto S, Russo V, Ozkul A, Sepici-Dincel A, Maiolino P, et al (2013) Bovine papillomavirus type 2 infects the urinary bladder of water buffalo (Bubalus bubalis) and plays a crucial role in the bubaline urothelial carcinogenesis. J Gen Virol 94: 403–408. 23100367 10.1099/vir.0.047662-0

[20] Shigehara K, Sasagawa T, Kawaguchi S, Nakashima T, Shimamura M, et al (2011) Etiologic role of human papillomavirus infection in bladder carcinoma. Cancer 117: 2067–2076. 21523718 10.1002/cncr.25777

[21] Stocco dos Santos RC, Lindsey CJ, Ferraz OP, Pinto JR, Mirandola RS, et al (1998) Bovine papillomavirus transmission and chromosomal aberrations: an experimental model. J Gen Virol 79: 2127–35. 9747721 10.1099/0022-1317-79-9-2127

[22] Roperto S, Borzacchiello G, Brun R, Leonardi L, Maiolino P, et al (2010) Review of bovine urothelial tumours and tumour-like lesions of the urinary bladder. J Comp Pathol 142: 95–108. 19818448 10.1016/j.jcpa.2009.08.156

[23] Griesser H, Sander H, Walczak C, Hilfrich RA (2009) HPV vaccine protein L1 predicts disease outcome of high-risk HPV+ early squamous dysplastic lesions. Am J Clin Pathol 132: 840–845. 19926574 10.1309/AJCPCU0HBFFFGDTV

[24] Bodily J, Laimins LA (2011) Persistence of human papillomavirus infection: keys to malignant progression. Trends in Microbiol 19: 33–39. 10.1016/j.tim.2010.10.002 PMC3059725 21050765

[25] Middleton K, Peh W, Southern S, Griffin H, Sotlar K, et al (2003) Organization of human papillomavirus productive cycle during neoplastic progression provides a basis for selection of diagnostic markers. J Virol 77: 10186–10201. 12970404 10.1128/JVI.77.19.10186-10201.2003 PMC228472

[26] zur Hausen H (2002) Papillomavirus and cancer: from basic studies to clinical application. Nat Rev Cancer 2: 342–350. 12044010 10.1038/nrc798

[27] Pett M, Coleman N (2007) Integration of high-risk human papillomavirus: a key event in cervical carcinogenesis? J Pathol 212: 356–367. 17573670 10.1002/path.2192

[28] Doorbar J (2007) Papillomavirus life cycle organization and biomarker selection. Dis Markers 23: 297–313. 17627064 10.1155/2007/613150 PMC3851388

[29] Häfner N, Driesch C, Gajda M, Jansen L, Kirchmayr R, et al (2008) Integration of the HPV16 genome does not invariably result in high levels of viral oncogene transcripts. 27: 1610–1617. 10.1038/sj.onc.1210791 17828299

[30] Das Ghosh D, Bhattacharrjee B, Sen S, Premi L, Mukhopadhyay I, et al (2012) Some novel insights on HPV16 related cervical cancer pathogenesis based on analyses of LCR methylation, viral load, E7 and E2/E4 expressions. PLoS One 7 (9) e44678 doi:10.1371/journal.pone.0044678. 22970286 10.1371/journal.pone.0044678 PMC3435323

[31] Xiao W, Bian M, Ma L, Liu J, Chen Y, et al (2010) Immunochemical analysis of human papillomavirus L1 capsid protein in liquid-based cytology samples from cervical lesions. Acta Cytol 54: 661–667. 20968152 10.1159/000325229

[32] Yemelyanova A, Gravitt PE, Ronnett BM, Rositch AF, Ogurtsova A, et al (2013) Immunohistochemical detection of human papillomavirus capsid proteins L1 and L2 in squamous intraepithelial lesions: potential utility in diagnosis and management. Mod Pathol 26: 268–274. 22996373 10.1038/modpathol.2012.156 PMC3530630

[33] Sarmadi S, Izadi-mood N, Pourlashkari M, Yarandi F, Sanii S (2010) HPV L1 capsid protein expression in squamous intraepithelial lesions of cervix uteri and its relevance to disease outcome. Arch Gynecol Obstet 285: 779–784. 10.1007/s00404-011-2010-y 21789516

[34] Hernandez J, Elahi A, Siegel E, Coppola D, Riggs B, et al (2011) HPV L1 capsid protein detection and progression of anal squamous neoplasia. Am J Clin Pathol 135: 436–441. 21350099 10.1309/AJCPR5VD6NSQRWBN PMC4511275

[35] Melsheimer P, Kaul S, Dobeck S, Bastert G (2003) Immunocytochemical detection of HPV high-risk type L1 capsid proteins in LSIL and HSIL as compared with detection of HPV L1 DNA. Acta Cytol 47: 124–128. 12685176 10.1159/000326491

[36] Shigehara K, Sasagawa T, Doorbar J, Kawaguchi S, Kobori Y, et al (2011) Etiological role of human papillomavirus infection for inverted papilloma of the bladder. J Med Virol 83: 277–285. 21181923 10.1002/jmv.21966

[37] Campo MS, Jarrett WFH, Barron RJ, O'Neil BW, Smith KT (1992) Association of bovine papillomavirus type 2 and bracken fern with bladder cancer in cattle. Cancer Res 53: 1–7. 1333885

[38] Wosiaki SR, Claus MP, Alfieri AL, Alfieri AA (2005) Bovine papillomavirus type 2 detection in the urinary bladder of cattle with chronic enzootic haematuria. Mem Inst Oswaldo Cruz 101: 635–638. 10.1590/s0074-02762006000600009 17072475

[39] Morgan IM, Donaldson MM (2006) The papillomavirus transcription/replication factor E2: structure, function, cancer and therapy. In: Campo MS, editor. Papillomavirus Research - from Natural History to Vaccines and Beyond, pp 73–82, Norfolk, UK:Caister Academic Press.

[40] Campo MS (2002) Animal model of papillomavirus pathogenesis. Virus Res 89: 249–261. 12445664 10.1016/s0168-1702(02)00193-4

[41] Alvarez J, Pokomandy ADE, Rouleau D, Ghattas G, Vezina S, et al (2010) Episomal and integrated human papillomavirus type 16 loads and anal intraepithelial neoplasia in HIV-seropositive men. AIDS 24: 2355–2363. 20706109 10.1097/QAD.0b013e32833db9ea

[42] Modis Y, Trus BI, Harrison SC (2001) Atomic model of the papillomavirus capsid. EMBO J 21: 4754–4762. 10.1093/emboj/cdf494 PMC126290 12234916

[43] Roperto S, Di Guardo G, Leonardi L, Pagnini U, Manco E, et al (2012) Bacterial isolates from the urine of cattle affected by urothelial tumors of the urinary bladder. Res Vet Sci 93: 1361–1366. 22819732 10.1016/j.rvsc.2012.06.009

[44] Peek RM, Mohla S, DuBois RN (2005) Inflammation in the genesis and perpetuation of cancer: summary and recommendations from a National Cancer Institute-sponsored meeting. Cancer Res 65: 8583–8587. 16204020 10.1158/0008-5472.CAN-05-1777

